# Size-Dependent Free Vibration and Buckling of Three-Dimensional Graphene Foam Microshells Based on Modified Couple Stress Theory

**DOI:** 10.3390/ma12050729

**Published:** 2019-03-02

**Authors:** Yunfei Liu, Yanqing Wang

**Affiliations:** 1 Department of Mechanics, Northeastern University, Shenyang 110819, China; lyfboook@163.com; 2 Key Laboratory of Ministry of Education on Safe Mining of Deep Metal Mines, Northeastern University, Shenyang 110819, China

**Keywords:** three-dimensional graphene foam microshell, vibration, buckling, Love’s thin shell theory, modified couple stress theory, size effect

## Abstract

In this research, the vibration and buckling of three-dimensional graphene foam (3D-GrF) microshells are investigated for the first time. In the microshells, three-dimensional graphene foams can distribute uniformly or non-uniformly through the thickness direction. Based on Love’s thin shell theory and the modified couple stress theory (MCST), size-dependent governing equations and corresponding boundary conditions are established through Hamilton’s principle. Then, vibration and axial buckling of 3D-GrF microshells are analyzed by employing the Navier method and Galerkin method. Results show that the graphene foam distribution type, size effect, the foam coefficient, the radius-to-thickness ratio, and the length-to-radius ratio play important roles in the mechanical characteristics of 3D-GrF microshells.

## 1. Introduction

Three-dimensional graphene foams (3D-GrFs) [1,2,3], unlike conventional polymeric open-cell foam materials, are a very new kind of nanofoam materials with three-dimensionally interconnected constituent graphene flakes. These 3D-GrFs have been synthesized by some approaches such as hard templating [4], sol–gel reaction [5], solution processing [6], powder metallurgy [7], 3D printing [8], freeze drying [9], hydrothermal reduction [10], and chemical vapor deposition [11]. These 3D-GrFs possess excellent properties of good electrical conductivity, high energy dissipation, super low density, superelasticity, and electrochemical stability [12,13,14,15]. These combined properties enable 3D-GrFs to be applied in electronics and energy storage/conversion systems [16,17], gas detection [15], sorbent materials [18], stretchable electronics [11], and so on.

In contrast to many experimental studies on the mechanical property of 3D-GrFs, few computational and theoretical investigations have been conducted to evaluate the relationship between the macro-mechanical characteristics and the intrinsic micro/nanostructures. Jinlong et al. [19] investigated and discussed the effects of graphene layer number and 3D-GrF defects on the electrical and mechanical properties of 3D-GrFs. Based on the coarse-grained molecular dynamics simulation, the constitutive relation between 3D-GrFs and microscopic deformation mechanisms were investigated by Wang et al. [20]. The mechanical properties in both tension and compression of 3D-GrFs at macro and nanoscales were evaluated by Nieto et al. [21]. Qin et al. [3] stated that 3D-GrFs have an exceptionally high tensile strength and they are 10 times as strong as mild steel. Nautiyal et al. [22] studied the dynamic mechanical properties of 3D-GrFs by nanoindentation technique.

As material size changes from macroscale to nano/microscale, size effect on mechanical properties of materials should be taken into account. It is reported that size effects in micro/nanostructures have been experimentally observed [23,24,25]. Due to the exclusion of size effect in the classical theory (CT), several non-classical theories were developed for analysis of micro/nanostructures. One of the non-classical theories incorporating size effect is the modified couple stress theory [26,27]. This theory has been employed in many aspects to interpret the size effect in micro/nanostructures [28,29,30,31,32,33,34].

Micro/nanoshells are an important and widely used form of micro/nanostructures in various engineering fields. Gholami et al. [35] analyzed the dynamic stability and axial buckling of functionally graded cylindrical microshells. Based on the strain gradient elasticity theory, Zhang et al. [36] developed a shear deformable functionally graded microshell model. Ghayesh and Farokhi [37] studied the nonlinear dynamical characteristics of doubly curved shallow microshells. SafarPour et al. [38] investigated the influences of various temperature distributions on the vibration of functionally graded rotating cylindrical microshells. Afterward, the buckling behavior of functionally graded sandwich microshells under axial loads was studied by Zeighampour and Shojaeian [39]. Wang et al. [40] studied nonlinear vibrations of cylindrical nanoshells conveying fluid in the framework of the surface stress elasticity theory.

To date, no work has been published on the mechanical properties of 3D-GrF microshells. In this paper, we aim to conduct size-dependent buckling and free vibration analysis of 3D-GrF cylindrical microshells. The couple stress theory and Love’s thin shell theory are used to derive the governing equations. After that, natural frequencies and critical buckling loads are solved by adopting the Navier method and Galerkin method. Finally, the results are illustrated for different parameters. 

## 2. Theoretical Formulation

### 2.1. Modified Couple Stress Theory

Based on the modified couple stress theory, the strain energy *U_s_* in a deformed elastic body occupying a volume *Ω* is [27]

(1) $$U_{s}=\frac{1}{2}\int_{\Omega }\left(m:\chi +\sigma :\varepsilon \right)dV$$

where **m** represents the deviatoric part of the couple stress tensor; χ is the symmetric curvature tensor; σ is the Cauchy stress tensor; ε is the strain tensor. These tensors are defined by [41]:

(2) σ=λ tr(ε)I+2με

(3) $$m=2l^{2}\mu \chi$$

(4) $$\varepsilon =\frac{1}{2}\left[\nabla u+{\left(\nabla u\right)}^{T}\right]$$

(5) $$\chi =\frac{1}{2}\left[\nabla \theta +{\left(\nabla \theta \right)}^{T}\right]$$

where *μ* and *λ* represent Lamé constants; u represents the displacement vector; *l* represent a material length scale parameter; θ is the rotation vector given by

(6) $$\theta =\frac{1}{2}\mathrm{curl}\;u.$$

The classical and higher-order strains are derived as [42,43]

(7) $$\begin{array}{l} \varepsilon_{\left(j\right)}^{\left(i\right)}=\varepsilon_{j}^{i}\sqrt{\frac{g_{\underline{ii}}}{g_{\underline{jj}}}}=\frac{1}{2}\sqrt{\frac{g_{\underline{ii}}}{g_{\underline{jj}}}}\{\left[{\left(\frac{u^{\left(i\right)}}{\sqrt{g_{\underline{ii}}}}\right)}_{,j}+\Gamma_{mj}^{i}\frac{u^{\left(m\right)}}{\sqrt{g_{\underline{mm}}}}\right]\\ +g_{nj}g^{im}\left[{\left(\frac{u^{\left(n\right)}}{\sqrt{g_{\underline{nn}}}}\right)}_{,m}+\Gamma_{qm}^{n}\frac{u^{\left(q\right)}}{\sqrt{g_{\underline{qq}}}}\right]\}, \end{array}$$

(8) $$\eta_{\left(i)\;(j\right)}^{\left(k\right)}=\eta_{i\;j}^{k}\sqrt{\frac{g_{\underline{kk}}}{g_{\underline{ii}}g_{\underline{jj}}}}=\frac{1}{2}\sqrt{\frac{g_{\underline{kk}}}{g_{\underline{ii}}g_{\underline{jj}}}}\left(u_{;ij}^{k}+u_{;ji}^{k}\right),$$

(9) $$\begin{array}{l} u_{;lm}^{k}={\left(\frac{u^{\left(k\right)}}{\sqrt{g_{\underline{kk}}}}\right)}_{,lm}+\Gamma_{ql}^{k}{\left(\frac{u^{\left(q\right)}}{\sqrt{g_{\underline{qq}}}}\right)}_{,m}+\Gamma_{qm}^{k}{\left(\frac{u^{\left(q\right)}}{\sqrt{g_{\underline{qq}}}}\right)}_{,l}-\Gamma_{ml}^{q}{\left(\frac{u^{\left(k\right)}}{\sqrt{g_{\underline{kk}}}}\right)}_{,q}\\ +\left[{\left(\Gamma_{lp}^{k}\right)}_{,m}+\Gamma_{qm}^{k}\Gamma_{pl}^{q}-\Gamma_{pq}^{k}\Gamma_{ml}^{q}\right]\times \frac{u^{\left(p\right)}}{\sqrt{g_{\underline{pp}}}}, \end{array}$$

where $\eta_{\left(i)\;(j\right)}^{\left(k\right)}$, $\varepsilon_{\left(j\right)}^{\left(i\right)}$, and *u*^(*k*)^ represent the physical components of higher-order displacement gradient $\eta_{i\;j}^{k}$, displacement gradient $\varepsilon_{j}^{i}$, and displacement vector *u^k^*, respectively; *g_ii_* and $\Gamma_{jk}^{i}$ denote an individual diagonal covariant component of the Euclidean metric tensor and Christoffel symbols of the second kind, respectively. The underscores under the indices denote no summation over indices. In cylindrical coordinates, the components of metric tensor and Christoffel symbols are expressed as

(10) $$\begin{array}{l} g_{\theta \theta }={\left[R\left(1+\frac{z}{R}\right)\right]}^{2},\;g_{xx}=1\;,\;g_{zz}=1,\;g_{kl}=0\;(k\neq l),\\ \Gamma_{\theta \theta }^{z}=-R\left(1+\frac{z}{R}\right),\;\Gamma_{\theta z}^{\theta }=\Gamma_{z\theta }^{\theta }={\left[R\left(1+\frac{z}{R}\right)\right]}^{-1}. \end{array}$$

Substituting Equations (9) and (10) into Equation (8) gives

(11) $$\begin{array}{l} \eta_{xxx}=\frac{\partial^{2}u}{\partial x^{2}},\;\eta_{xx\theta }=\frac{\partial^{2}v}{\partial x^{2}},\;\eta_{x\theta \theta }=\eta_{\theta x\theta }={\left[R\left(1+\frac{z}{R}\right)\right]}^{-1}\left[\frac{\partial^{2}v}{\partial x\partial \theta }+\frac{\partial w}{\partial x}\right],\\ \eta_{xxz}=\frac{\partial^{2}w}{\partial x^{2}},\;\eta_{zxx}=\eta_{xzx}=\frac{\partial^{2}u}{\partial z\partial x},\\ \eta_{zzz}=\frac{\partial^{2}w}{\partial z^{2}},\;\eta_{\theta xx}=\eta_{x\theta x}={\left[R\left(1+\frac{z}{R}\right)\right]}^{-1}\frac{\partial^{2}u}{\partial x\partial \theta },\\ \eta_{z\theta \theta }=\eta_{\theta z\theta }={\left[R\left(\frac{z}{R}+1\right)\right]}^{-1}\times \left\{\frac{\partial^{2}v}{\partial z\partial \theta }+\frac{\partial w}{\partial z}-{\left[R\left(1+\frac{z}{R}\right)\right]}^{-1}w-{\left[R\left(1+\frac{z}{R}\right)\right]}^{-1}\frac{\partial v}{\partial \theta }\right\},\\ \eta_{z\theta z}=\eta_{\theta zz}={\left[R\left(\frac{z}{R}+1\right)\right]}^{-1}\times \left\{\frac{\partial^{2}w}{\partial z\partial \theta }-\frac{\partial v}{\partial z}-{\left[R\left(1+\frac{z}{R}\right)\right]}^{-1}\frac{\partial w}{\partial \theta }-{\left[R\left(\frac{z}{R}+1\right)\right]}^{-1}v\right\},\\ \eta_{zxz}=\eta_{xzz}=\frac{\partial^{2}w}{\partial z\partial x},\;\eta_{zz\theta }=\frac{\partial^{2}v}{\partial z^{2}},\;\eta_{x\theta z}=\eta_{\theta xz}={\left[R\left(1+\frac{z}{R}\right)\right]}^{-1}\left(\frac{\partial^{2}w}{\partial x\partial \theta }-\frac{\partial v}{\partial x}\right),\\ \eta_{\theta \theta x}={\left[R\left(\frac{z}{R}+1\right)\right]}^{-1}\left\{{\left[R\left(1+\frac{z}{R}\right)\right]}^{-1}\frac{\partial^{2}u}{\partial \theta^{2}}+\frac{\partial u}{\partial z}\right\},\\ \eta_{\theta \theta \theta }={\left[R\left(\frac{z}{R}+1\right)\right]}^{-2}\left[\frac{\partial^{2}v}{\partial \theta^{2}}+2\frac{\partial w}{\partial \theta }+R\left(1+\frac{z}{R}\right)\frac{\partial v}{\partial z}-v\right],\\ \eta_{\theta \theta z}={\left[R\left(\frac{z}{R}+1\right)\right]}^{-2}\left[\frac{\partial^{2}w}{\partial \theta^{2}}-2\frac{\partial v}{\partial \theta }+R\left(1+\frac{z}{R}\right)\frac{\partial w}{\partial z}-w\right]. \end{array}$$

### 2.2. 3D-GrF Circular Cylindrical Microshell 

**Figure 1:**
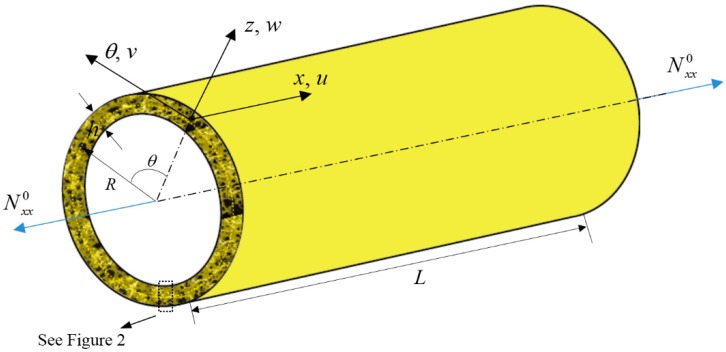
Schematic of a three-dimensional graphene foam (3D-GrF) circular cylindrical microshell.

In Figure 1, a 3D-GrF microshell with the thickness *h*, the middle-plane radius *R*, and the length *L* is shown. *u*(*x*, *θ*, *t*), *v*(*x*, *θ*, *t*), and *w*(*x*, *θ*, *t*) represent the in-plane and transverse displacements of points at the middle plane; $N_{xx}^{0}$ represents the axial load applied to the microshell. 

**Figure 2:**
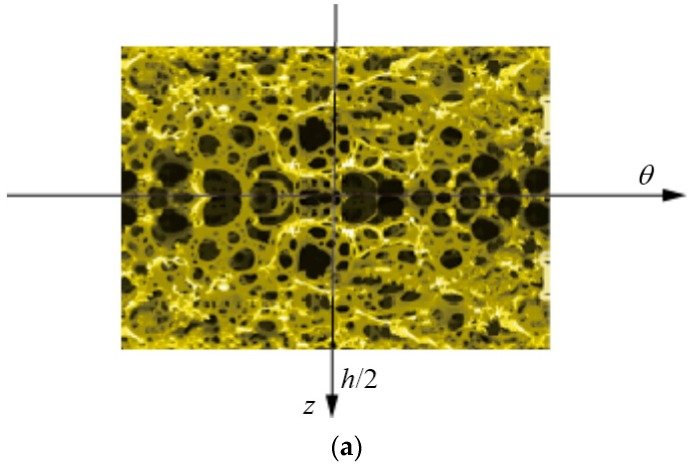

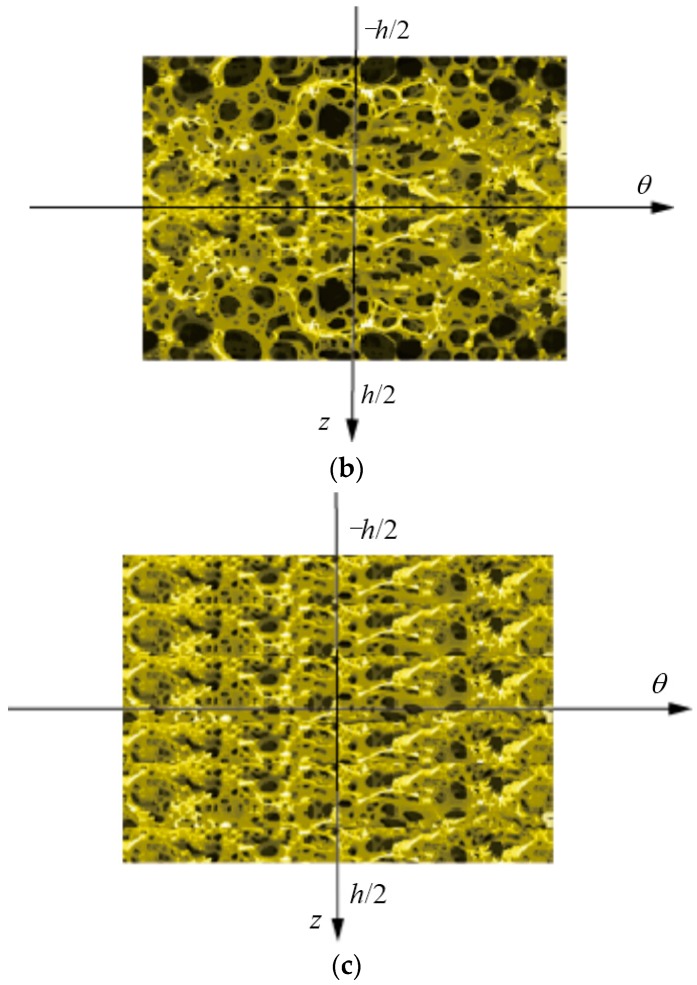
3D-GrF distributions in the thickness direction: (**a**) 3D-GrF-I; (**b**) 3D-GrF-II; (**c**) 3D-GrF-U.

Three types of foam distributions in the thickness direction were considered, as shown in Figure 2. Herein, Figure 2a,b depict non-uniform foam distribution while Figure 2c shows uniform foam distribution. They are denoted by 3D-GrF-I, 3D-GrF-II, and 3D-GrF-U, respectively. As shown in Figure 2, the largest foams are located on the mid-plane for 3D-GrF-I while on the top and bottom surfaces for 3D-GrF-II, leading to the variations of material properties given in Equations (12)–(14) for 3D-GrF-I, and Equations (15)–(17) for 3D-GrF-II. Material properties of 3D-GrF-U are described in Equations (18)–(20). They are given by [44,45,46]:

(12) $$E(z)=E_{1}\left[1-\kappa_{0}\cos(\pi z/h)\right]$$

(13) $$\rho (z)=\rho_{1}\left[1-\kappa_{m}\cos(\pi z/h)\right]$$

(14) $$G(z)=G_{1}\left[1-\kappa_{0}\cos(\pi z/h)\right]$$

(15) $$E(z)=E_{1}\left\{1-\kappa_{0}^{*}\left[1-\cos(\pi z/h)\right]\right\}$$

(16) $$\rho (z)=\rho_{1}\left\{1-\kappa_{m}^{*}\left[1-\cos(\pi z/h)\right]\right\}$$

(17) $$G(z)=G_{1}\left\{1-\kappa_{0}^{*}\left[1-\cos(\pi z/h)\right]\right\}$$

(18) $$E(z)=E_{1}\vartheta$$

(19) $$\rho (z)=\rho_{1}\vartheta^{\prime }$$

(20) $$G(z)=G_{1}\vartheta$$

where *E*(*z*), *ρ*(*z*), and *G*(*z*) are general Young’s modules, mass density, and shear modules of the 3D-GrF microshell, respectively; *E*_1_, *G*_1_, and *ρ*_1_ represent corresponding properties of solid graphenes without internal foams; $\kappa_{0}$ and $\kappa_{m}$ represent coefficients of foams and mass density for 3D-GrF-I, respectively; $\kappa_{0}^{*}$ and $\kappa_{m}^{*}$ are corresponding coefficients for 3D-GrF-II; ϑ and $\vartheta^{\prime }$ are corresponding coefficients for 3D-GrF-U. Thereinto, shear modulus *G*_1_ is calculated by

(21) $$G_{1}=\frac{E_{1}}{2\left(1+\nu \right)}$$

where ν represents Poisson’s ratio. 

The typical mechanical property of 3D-GrFs [3,20,21], shown in Equation (22), is employed to establish the relationships in Equation (23) between mass density coefficients and foam coefficients for different foam distributions:

(22) $$\frac{E\left(z\right)}{E_{1}}={\left[\frac{\rho \left(z\right)}{\rho_{1}}\right]}^{2.73}$$

(23) $$\left\{ \begin{array}{cc} 1-\kappa_{m}\cos\left(\pi z/h\right)=\sqrt[{2.73}]{1-\kappa_{0}\cos\left(\pi z/h\right)} & 3D-\mathrm{GrF}-I\\ 1-\kappa_{m}^{*}\left[1-\cos\left(\pi z/h\right)\right]=\sqrt[{2.73}]{1-\kappa_{0}^{*}\left[1-\cos\left(\pi z/h\right)\right]} & 3D-\mathrm{GrF}-\mathrm{II}\\ \vartheta^{\prime }=\sqrt[{2.73}]{\vartheta } & 3D-\mathrm{GrF}-U \end{array}\right.$$

The masses of all 3D-GrF microshells with varying foams are set to be equivalent, namely

(24) $$\{\begin{array}{c} \int_{0}^{h/2}\sqrt[{2.73}]{1-\kappa_{0}^{*}\left[1-\cos\left(\pi z/h\right)\right]}dz=\int_{0}^{h/2}\sqrt[{2.73}]{1-\kappa_{0}\cos\left(\pi z/h\right)}dz\\ \int_{0}^{h/2}\sqrt[{2.73}]{\vartheta }dz=\int_{0}^{h/2}\sqrt[{2.73}]{1-\kappa_{0}\cos\left(\pi z/h\right)}dz \end{array}$$

which can be used to determine $\kappa_{0}^{*}$ and ϑ with a given value of $\kappa_{0}$, as tabulated in Table 1. It is seen that $\kappa_{0}^{*}$ rises dramatically with the increase of $\kappa_{0}$. When $\kappa_{0}$ reaches 0.65, $\kappa_{0}^{*}$ is close to the upper limit ($\kappa_{0}^{*}=0.9976$). Therefore, the selected range of $\kappa_{0}\in$ [0, 0.65] is applied in the following numerical calculations. 

The displacement fields, based on Love’s thin shell theory, are expressed as [47]

(25) $$u_{1}(x,\theta ,z,t)=u(x,\theta ,t)-z\frac{\partial w(x,\theta ,t)}{\partial x}$$

(26) $$u_{2}(x,\theta ,z,t)=v(x,\theta ,t)-\frac{z}{R}\left[\frac{\partial w(x,\theta ,t)}{\partial \theta }-v(x,\theta ,t)\right]$$

(27) $$u_{3}(x,\theta ,z,t)=w(x,\theta ,t)$$

where *t* is time, and $u_{1}(x,\theta ,z,t)$, $u_{2}(x,\theta ,z,t)$, and $u_{3}(x,\theta ,z,t)$ are displacements of an arbitrary point of the microshell along *x*-, θ-, and *z*-axes, respectively.

By substituting Equations (25)–(27) and Equation (10) into Equation (7), strain–displacement relations are expressed as:

(28) $$\varepsilon_{xx}=\frac{\partial u}{\partial x}-z\frac{\partial^{2}w}{\partial x^{2}}$$

(29) $$\varepsilon_{\theta \theta }=\frac{1}{R}\frac{\partial v}{\partial \theta }+\frac{w}{R}-\frac{z}{R^{2}}\frac{\partial^{2}w}{\partial \theta^{2}}$$

(30) $$\gamma_{x\theta }=\frac{\partial v}{\partial x}+\frac{1}{R}\frac{\partial u}{\partial \theta }-\frac{2z}{R}\frac{\partial^{2}w}{\partial \theta \partial x}.$$

Nonzero components of χ can be obtained by substituting Equations (25)–(27) into Equation (11) and using Equation (5):

(31) $$\chi_{xx}=-\frac{1}{R}\left(\frac{\partial v}{\partial x}-\frac{\partial^{2}w}{\partial x\partial \theta }\right)$$

(32) $$\chi_{zz}=\frac{1}{2R^{2}}\left(\frac{\partial u}{\partial \theta }-2z\frac{\partial^{2}w}{\partial x\partial \theta }+R\frac{\partial v}{\partial x}\right)$$

(33) $$\chi_{\theta \theta }=\frac{1}{2R}\left(\frac{\partial v}{\partial x}-2\frac{\partial^{2}w}{\partial x\partial \theta }-\frac{1}{R}\frac{\partial u}{\partial \theta }\right)$$

(34) $$\chi_{x\theta }=\chi_{\theta x}=\frac{1}{2}\left(-\frac{\partial^{2}w}{\partial x^{2}}-\frac{1}{R^{2}}\frac{\partial v}{\partial \theta }+\frac{1}{R^{2}}\frac{\partial^{2}w}{\partial \theta^{2}}\right)$$

(35) $$\chi_{xz}=\chi_{zx}=\frac{1}{4}\left(\frac{\partial^{2}v}{\partial x^{2}}-\frac{1}{R}\frac{\partial^{2}u}{\partial x\partial \theta }-\frac{z^{2}}{R^{2}}\frac{\partial^{3}w}{\partial x^{2}\partial \theta }\right)$$

(36) $$\chi_{z\theta }=\chi_{\theta z}=\frac{1}{4R}\left(-\frac{1}{R}\frac{\partial^{2}u}{\partial \theta^{2}}-\frac{z^{2}}{R^{2}}\frac{\partial^{3}w}{\partial x\partial \theta^{2}}+\frac{\partial^{2}v}{\partial x\partial \theta }+2\frac{\partial w}{\partial x}\right).$$

According to Equation (1), the strain energy can be written as

(37) $$\begin{array}{ll} U_{s}= & \frac{1}{2}\int_{0}^{L}\int_{0}^{2\pi }[N_{xx}\frac{\partial u}{\partial x}+\left(\frac{N_{x\theta }}{R}-\frac{Y_{\theta \theta }}{2R^{2}}+\frac{Y_{zz}}{2R^{2}}\right)\frac{\partial u}{\partial \theta }-\frac{Y_{z\theta }}{2R^{2}}\frac{\partial^{2}u}{\partial \theta^{2}}\\ & -\frac{Y_{zx}}{2R}\frac{\partial^{2}u}{\partial x\partial \theta }+\left(N_{x\theta }-\frac{Y_{xx}}{R}+\frac{Y_{zz}}{2R}+\frac{Y_{\theta \theta }}{2R}\right)\frac{\partial v}{\partial x}+\frac{Y_{zx}}{2}\frac{\partial^{2}v}{\partial x^{2}}+\frac{Y_{z\theta }}{R}\frac{\partial w}{\partial x}\\ & +\left(\frac{N_{\theta \theta }}{R}-\frac{Y_{x\theta }}{R^{2}}\right)\frac{\partial v}{\partial \theta }+\frac{Y_{z\theta }}{2R}\frac{\partial^{2}v}{\partial x\partial \theta }+\left(Y_{xx}-M_{xx}-Y_{x\theta }\right)\frac{\partial^{2}w}{\partial x^{2}}\\ & +\left(Y_{\theta \theta }-\frac{M_{\theta \theta }}{R^{2}}+\frac{Y_{x\theta }}{R^{2}}\right)\frac{\partial^{2}w}{\partial \theta^{2}}+\left(Y_{x\theta }+Y_{z\theta }-\frac{2M_{x\theta }}{R}+\frac{Y_{xx}}{R}-\frac{Y_{\theta \theta }}{R}-\frac{T_{zz}}{R^{2}}\right)\frac{\partial^{2}w}{\partial x\partial \theta }\\ & +\frac{N_{\theta \theta }}{R}w]Rd\theta dx \end{array}$$

The non-classical and classical moments and forces are given by

(38) $$\begin{array}{ll} T_{ij}=\int_{-\frac{h}{2}}^{\frac{h}{2}}m_{ij}zdz, & Y_{ij}=\int_{-\frac{h}{2}}^{\frac{h}{2}}m_{ij}dz,\\ M_{ij}=\int_{-\frac{h}{2}}^{\frac{h}{2}}\sigma_{ij}zdz, & N_{ij}=\int_{-\frac{h}{2}}^{\frac{h}{2}}\sigma_{ij}dz. \end{array}$$

The kinetic energy of the microshell is

(39) $$T=\frac{1}{2}\int_{V}\rho (z)\left[{\left(\frac{\partial u}{\partial t}-z\frac{\partial^{2}w}{\partial x\partial t}\right)}^{2}+{\left(\frac{\partial v}{\partial t}-\frac{z}{R}\frac{\partial^{2}w}{\partial \theta \partial t}\right)}^{2}+{\left(\frac{\partial w}{\partial t}\right)}^{2}\right]dV.$$

The work performed by axial load $N_{xx}^{0}$ applied on the middle surface of the 3D-GrF microshell is given by [48]

(40) $$W_{F}=\int_{A}\left[\frac{1}{2}N_{xx}^{0}\cdot {\left(\frac{\partial w}{\partial x}\right)}^{2}\right]dA.$$

Using Hamilton’s principle [49,50,51,52,53,54]

(41) $$\int_{0}^{t}\left(\delta U_{s}-\delta T-\delta W_{F}\right)dt=0$$

and applying Equations (37), (39) and (40) in Equation (41), the governing equations of motion for the 3D-GrF cylindrical microshell are

(42) $$\begin{array}{l} -D_{1,0}\frac{\partial^{2}u}{\partial x^{2}}+\frac{D_{5,0}l^{2}}{4R^{2}}\frac{\partial^{4}u}{\partial x^{2}\partial \theta^{2}}-\frac{D_{5,0}l^{2}}{4R}\frac{\partial^{4}v}{\partial x^{3}\partial \theta }-\frac{1}{R}\left(D_{3,0}+D_{5,0}\right)\frac{\partial^{2}v}{\partial x\partial \theta }-\frac{D_{5,0}l^{2}}{4R^{3}}\frac{\partial^{4}v}{\partial x\partial \theta^{3}}\\ \;-\frac{1}{R}D_{3,0}\frac{\partial w}{\partial x}+\left(\frac{1}{R^{2}}D_{3,1}+\frac{2}{R^{2}}D_{5,1}+\frac{l^{2}}{R^{4}}D_{5,1}-\frac{3l^{2}}{2R^{3}}D_{5,0}\right)\frac{\partial^{3}w}{\partial x\partial \theta^{2}}\\ \;-\frac{D_{5,0}}{R^{2}}\left(1+\frac{l^{2}}{R}\right)\frac{\partial^{2}u}{\partial \theta^{2}}+\frac{D_{5,0}l^{2}}{4R^{4}}\frac{\partial^{4}u}{\partial \theta^{4}}+D_{1,1}\frac{\partial^{3}w}{\partial x^{3}}-I_{1,1}\frac{\partial^{3}w}{\partial x\partial t^{2}}+I_{1,0}\frac{\partial^{2}u}{\partial t^{2}}=0 \end{array}$$

(43) $$\begin{array}{l} \frac{l^{2}}{4}D_{5,0}\frac{\partial^{4}v}{\partial x^{4}}-D_{5,0}\left(1+\frac{3l^{2}}{R^{2}}\right)\frac{\partial^{2}v}{\partial x^{2}}-\frac{1}{R^{2}}\left(D_{1,0}+\frac{l^{2}}{R^{2}}D_{5,0}\right)\frac{\partial^{2}v}{\partial \theta^{2}}+\frac{l^{2}}{4R^{2}}D_{5,0}\frac{\partial^{4}v}{\partial x^{2}\partial \theta^{2}}\\ \;-\frac{l^{2}}{4R}D_{5,0}\frac{\partial^{4}u}{\partial x^{3}\partial \theta }-\frac{l^{2}}{4R^{3}}D_{5,0}\frac{\partial^{4}u}{\partial x\partial \theta^{3}}-\frac{1}{R}\left(D_{3,0}+D_{5,0}\right)\frac{\partial^{2}u}{\partial x\partial \theta }\\ \;+\left(\frac{1}{R}D_{3,1}+\frac{2}{R}D_{5,1}+\frac{l^{2}}{R^{3}}D_{5,1}+D_{5,0}\frac{5l^{2}}{2R^{2}}\right)\frac{\partial^{3}w}{\partial x^{2}\partial \theta }+\frac{l^{2}}{R^{4}}D_{5,0}\frac{\partial^{3}w}{\partial \theta^{3}}\\ \;-\frac{1}{R^{2}}D_{1,0}\frac{\partial w}{\partial \theta }+\frac{1}{R^{3}}D_{1,1}\frac{\partial^{3}w}{\partial \theta^{3}}-\frac{1}{R}I_{1,1}\frac{\partial^{3}w}{\partial \theta \partial t^{2}}+I_{1,0}\frac{\partial^{2}v}{\partial t^{2}}=0 \end{array}$$

(44) $$\begin{array}{l} \left(D_{1,2}+l^{2}D_{5,0}\right)\frac{\partial^{4}w}{\partial x^{4}}-\left(\frac{2}{R}D_{3,1}+D_{5,0}\frac{l^{2}}{R^{2}}\right)\frac{\partial^{2}w}{\partial x^{2}}+\frac{1}{R}D_{3,0}\frac{\partial u}{\partial x}+\frac{1}{R^{2}}D_{1,0}w\\ \;+\frac{1}{R^{2}}\left(2D_{3,2}+4D_{5.2}+\frac{l^{2}}{R^{2}}D_{5.2}+2l^{2}D_{5.0}\right)\frac{\partial^{4}w}{\partial x^{2}\partial \theta^{2}}+\frac{1}{R^{2}}D_{1,0}\frac{\partial v}{\partial \theta }\\ \;-\frac{1}{R^{2}}\left(D_{3,1}+2D_{5,1}+\frac{l^{2}}{R^{2}}D_{5.1}-D_{5.0}\frac{3l^{2}}{2R}\right)\frac{\partial^{3}u}{\partial x\partial \theta^{2}}-\left(\frac{1}{R^{3}}D_{1,1}+\frac{l^{2}}{R^{4}}D_{5.0}\right)\frac{\partial^{3}v}{\partial \theta^{3}}\\ \;-\frac{1}{R}\left(D_{3,1}+2D_{5,1}+\frac{l^{2}}{R^{2}}D_{5.1}+D_{5.0}\frac{5l^{2}}{2R}\right)\frac{\partial^{3}v}{\partial x^{2}\partial \theta }+\frac{1}{R^{4}}\left(D_{1,2}+l^{2}D_{5.0}\right)\frac{\partial^{4}w}{\partial \theta^{4}}\\ \;-D_{1,1}\frac{\partial^{3}u}{\partial x^{3}}-\frac{2}{R^{3}}D_{1,1}\frac{\partial^{2}w}{\partial \theta^{2}}-I_{1,2}\frac{\partial w}{\partial x^{2}\partial t^{2}}-\frac{1}{R^{2}}I_{1,2}\frac{\partial^{4}w}{\partial \theta^{2}\partial t^{2}}+I_{1,1}\frac{\partial^{3}u}{\partial x\partial t^{2}}\\ \;+\frac{1}{R}I_{1,1}\frac{\partial^{3}v}{\partial \theta \partial t^{2}}+I_{1,0}\frac{\partial^{2}w}{\partial t^{2}}+N_{xx}^{0}\frac{\partial^{2}w}{\partial x^{2}}=0 \end{array}$$

where

$$D_{1,i}=\int_{-\frac{h}{2}}^{\frac{h}{2}}\frac{E(z)}{1-\nu^{2}}\;z^{i}dz\;(i=0,1,2)$$

$$D_{3,i}=\int_{-\frac{h}{2}}^{\frac{h}{2}}\frac{E(z)\nu }{1-\nu^{2}}\;z^{i}dz\;(i=0,1,2)$$

$$D_{5,i}=\int_{-\frac{h}{2}}^{\frac{h}{2}}\mu (z)\;z^{i}dz\;(i=0,1,2)$$

$$I_{1,i}=\int_{-\frac{h}{2}}^{\frac{h}{2}}\rho (z)\;z^{i}dz\;(i=0,1,2).$$

Boundary conditions at the edges with *x* = constant are

(45) $$\begin{array}{l} \delta u_{x=0,L}=0\;\mathrm{or}\;\int_{\theta }[D_{1,0}\frac{\partial u}{\partial x}-\frac{D_{5,0}l^{2}}{4R^{2}}\frac{\partial^{3}u}{\partial x\partial \theta^{2}}+\frac{D_{5,0}l^{2}}{4R}\frac{\partial^{3}v}{\partial x^{2}\partial \theta }+\\ \frac{1}{R}D_{3,0}\left(\frac{\partial v}{\partial \theta }+w\right)-D_{1,1}\frac{\partial^{2}w}{\partial x^{2}}-\frac{1}{R^{2}}D_{3,1}\frac{\partial^{2}w}{\partial \theta^{2}}]{d\theta |}_{x=0,L}=0 \end{array}$$

(46) $$\begin{array}{l} \delta v_{x=0,L}=0\;\mathrm{or}\;\int_{\theta }[-\frac{D_{5,0}l^{2}}{4}\frac{\partial^{3}v}{\partial x^{3}}+\left(1+\frac{3l^{2}}{R^{2}}\right)D_{5,0}\frac{\partial v}{\partial x}-\frac{D_{5,0}l^{2}}{4R^{2}}\frac{\partial^{3}v}{\partial x\partial \theta^{2}}\\ \;+\frac{D_{5,0}l^{2}}{4R}\frac{\partial^{3}u}{\partial x^{2}\partial \theta }+\frac{D_{5,0}l^{2}}{4R^{3}}\frac{\partial^{3}u}{\partial \theta^{3}}+\frac{D_{5,0}}{R}\frac{\partial u}{\partial \theta }\\ \;-\left(\frac{7l^{2}D_{5,0}}{2R^{2}}+\frac{2D_{5,1}}{R}+\frac{l^{2}D_{5,1}}{R^{3}}\right)\frac{\partial^{2}w}{\partial x\partial \theta }]{d\theta |}_{x=0,L}=0 \end{array}$$

(47) $$\delta {\left(\frac{\partial v}{\partial x}\right)|}_{x=0,L}=0\;\mathrm{or}\;\int_{\theta }\left(\frac{D_{5,0}l^{2}}{4}\frac{\partial^{2}v}{\partial x^{2}}-\frac{D_{5,0}l^{2}}{4R}\frac{\partial^{2}u}{\partial x\partial \theta }\right){d\theta |}_{x=0,L}=0$$

(48) $$\begin{array}{l} \delta {w|}_{x=0,L}=0\;\mathrm{or}\;\int_{\theta }[-(D_{1,2}+D_{5,0}l^{2})\frac{\partial^{3}w}{\partial x^{3}}+\left(\frac{D_{5,0}l^{2}}{R^{2}}\right)\frac{\partial w}{\partial x}\\ -\left(\frac{1}{R^{2}}D_{3,2}+\frac{4D_{5,2}}{R^{2}}+\frac{3D_{5,0}l^{2}}{R^{2}}+\frac{2D_{5,2}l^{2}}{R^{4}}\right)\frac{\partial^{3}w}{\partial x\partial \theta^{2}}+D_{1,1}\frac{\partial^{2}u}{\partial x^{2}}\\ +\left(\frac{5D_{5,0}l^{2}}{2R^{2}}+\frac{1}{R}D_{3,1}+\frac{2}{R}D_{5,1}+\frac{l^{2}}{R}D_{5,1}\right)\frac{\partial^{2}v}{\partial x\partial \theta }\\ +\left(\frac{2}{R^{2}}D_{5,1}+\frac{l^{2}}{R^{4}}D_{5,1}-\frac{3D_{5,0}l^{2}}{2R^{3}}\right)\frac{\partial^{2}u}{\partial \theta^{2}}]{d\theta |}_{x=0,L}=0 \end{array}$$

(49) $$\begin{array}{l} \delta {\left(\frac{\partial w}{\partial x}\right)|}_{x=0,L}=0\;\mathrm{or}\;\int_{\theta }[(D_{1,2}+D_{5,0}l^{2})\frac{\partial^{2}W}{\partial x^{2}}+\left(\frac{D_{3,2}}{R^{2}}-\frac{D_{5,0}l^{2}}{R^{2}}\right)\frac{\partial^{2}w}{\partial \theta^{2}}\\ +\left(\frac{D_{5,0}l^{2}}{R^{2}}-\frac{1}{R}D_{3,1}\right)\frac{\partial v}{\partial \theta }-D_{1,1}\frac{\partial u}{\partial x}-\frac{1}{R}D_{3,1}w]{d\theta |}_{x=0,L}=0 \end{array}$$

Boundary conditions at the edges with *θ* = constant are:

(50) $$\begin{array}{l} \delta {u|}_{\theta =0,\theta_{0}}=0\;\mathrm{or}\;\int_{x}[\frac{D_{5,0}}{R^{2}}\left(1+\frac{l^{2}}{R^{2}}\right)\frac{\partial u}{\partial \theta }-\frac{D_{5,0}l^{2}}{4R^{2}}\frac{\partial^{3}u}{\partial x^{2}\partial \theta }+\frac{D_{5,0}l^{2}}{4R^{3}}\frac{\partial^{3}v}{\partial x\partial \theta^{2}}\\ +\frac{D_{5,0}}{R}\frac{\partial v}{\partial x}+\left(\frac{3D_{5,0}l^{2}}{2R^{3}}-\frac{2D_{5,1}}{R^{2}}-\frac{l^{2}D_{5,1}}{R^{4}}\right)\frac{\partial^{2}w}{\partial x\partial \theta }]{dx|}_{\theta =0,\theta_{0}}=0 \end{array}$$

(51) $$\begin{array}{l} \delta {v|}_{\theta =0,\theta_{0}}=0\;\mathrm{or}\;\int_{x}[\left(\frac{1}{R^{2}}D_{1,0}+\frac{D_{5,0}l^{2}}{R^{4}}\right)\frac{\partial v}{\partial \theta }+\frac{1}{R}D_{3,0}\frac{\partial u}{\partial x}\\ +\frac{1}{R^{2}}D_{1,0}w+\left(\frac{D_{5,0}l^{2}}{2R^{2}}-\frac{1}{R}D_{3,1}\right)\frac{\partial^{2}w}{\partial x^{2}}-\frac{D_{5,0}l^{2}}{4R^{2}}\frac{\partial^{3}v}{\partial x^{2}\partial \theta }+\frac{D_{5,0}l^{2}}{4R^{3}}\frac{\partial^{3}u}{\partial x\partial \theta^{2}}\\ -\left(\frac{D_{5,0}l^{2}}{R^{4}}+\frac{1}{R^{3}}D_{1,1}\right)\frac{\partial^{2}w}{\partial \theta^{2}}]{dx|}_{\theta =0,\theta_{0}}=0 \end{array}$$

(52) $$\delta {\left(\frac{\partial u}{\partial \theta }\right)|}_{\theta =0,\theta_{0}}=0\;\mathrm{or}\;\int_{x}\left(\frac{D_{5,0}l^{2}}{4R^{4}}\frac{\partial^{2}u}{\partial \theta^{2}}-\frac{D_{5,0}l^{2}}{4R^{3}}\frac{\partial^{2}v}{\partial x\partial \theta }-\frac{D_{5,0}l^{2}}{2R^{3}}\frac{\partial w}{\partial x}\right){dx|}_{\theta =0,\theta_{0}}=0$$

(53) $$\begin{array}{l} \delta {w|}_{\theta =0,\theta_{0}}=0\;\mathrm{or}\;\int_{x}[-\left(\frac{1}{R^{4}}D_{1,2}+\frac{D_{5,0}l^{2}}{R^{4}}\right)\frac{\partial^{3}w}{\partial \theta^{3}}+\frac{3D_{5,0}l^{2}}{R^{2}}\frac{\partial^{2}v}{\partial x^{2}}\\ -\left(\frac{1}{R^{2}}D_{3,2}+\frac{4D_{5,2}}{R^{2}}+\frac{3D_{5,0}l^{2}}{R^{2}}+\frac{2D_{5,2}l^{2}}{R^{4}}\right)\frac{\partial^{3}w}{\partial x^{2}\partial \theta }\\ +\left(\frac{D_{5,0}l^{2}}{R^{4}}+\frac{1}{R^{3}}D_{1,1}\right)\frac{\partial^{2}v}{\partial \theta^{2}}+\left(\frac{1}{R^{2}}D_{3,1}-\frac{D_{5,0}l^{2}}{R^{3}}\right)\frac{\partial^{2}u}{\partial x\partial \theta }\\ +\frac{1}{R^{3}}D_{1,1}\frac{\partial w}{\partial \theta }]{dx|}_{\theta =0,\theta_{0}}=0 \end{array}$$

(54) $$\begin{array}{l} \delta {\left(\frac{\partial w}{\partial \theta }\right)|}_{\theta =0,\theta_{0}}=0\;\mathrm{or}\;\int_{x}[\left(\frac{1}{R^{4}}D_{1,2}+\frac{1}{R^{4}}D_{5,0}l^{2}\right)\frac{\partial^{2}w}{\partial \theta^{2}}+\left(\frac{D_{3,2}}{R^{2}}-\frac{D_{5,0}l^{2}}{R^{2}}\right)\frac{\partial^{2}w}{\partial x^{2}}\\ -\left(\frac{D_{5,0}l^{2}}{R^{4}}+\frac{1}{R^{3}}D_{1,1}\right)\frac{\partial v}{\partial \theta }-\frac{1}{R^{2}}D_{3,1}\frac{\partial u}{\partial x}-\frac{1}{R^{3}}D_{1,1}w]{dx|}_{\theta =0,\theta_{0}}=0 \end{array}$$

## 3. Free Vibration and Buckling Analysis

### 3.1. Navier Solution

For simply supported–simply supported (SS–SS) 3D-GrF cylindrical microshells, the displacement functions using the Navier method can be expressed as:

(55) $$u(x,\theta ,t)=U_{mn}\cos\left(\frac{m\pi x}{L}\right)\cos(n\theta )e^{i\omega t}$$

(56) $$v(x,\theta ,t)=V_{mn}\sin\left(\frac{m\pi x}{L}\right)\sin(n\theta )e^{i\omega t}$$

(57) $$w(x,\theta ,t)=W_{mn}\sin\left(\frac{m\pi x}{L}\right)\cos(n\theta )e^{i\omega t}$$

where $U_{mn}$, $V_{mn}$, and $W_{mn}$ represent the displacement amplitude components; *m* and *n* are mode numbers; and *ω* is natural circular frequency of the 3D-GrF microshell. Obviously, displacement functions in Navier solution procedure satisfy the SS–SS boundary condition.

Substituting Equations (55)–(57) into Equations (42)–(44) and then removing trigonometric functions lead to

(58) $$\left(K+N_{xx}^{0}\cdot K_{g}\right)\cdot d+M\cdot \ddot{d}=\left\{\begin{array}{c} 0\\ 0\\ 0 \end{array}\right\}$$

where $d={\left[U_{mn},\;V_{mn},\;W_{mn}\right]}^{T}$; **K**, **K_g_**, and **M** denote stiffness matrix, geometric stiffness matrix, and mass matrix, respectively. The non-zero elements in the above matrices are given in the Appendix A.

Neglecting axial load $N_{xx}^{0}$, Equation (58) is reduced to the following eigenvalue problem of the 3D-GrF microshell:

(59) $$\left(K-\omega^{2}M\right)\cdot d=\left\{\begin{array}{c} 0\\ 0\\ 0 \end{array}\right\}.$$

If disregarding inertia terms and assuming $N_{xx}^{0}=-P$, Equation (58) is reduced to the equilibrium equations of a static buckling problem of the 3D-GrF microshell:

(60) $$\left(K-P\cdot K_{g}\right)\cdot d=\left\{\begin{array}{c} 0\\ 0\\ 0 \end{array}\right\}$$

where *P* represents buckling load. Therefore, the critical (minimum) buckling load *P*_cr_ and vibration frequencies of the 3D-GrF microshell are obtained by solving the above eigenvalue problems [55,56,57].

### 3.2. Galerkin Solution

In order to analyze free vibration of 3D-GrF microshells with other boundary conditions, a Galerkin-based solution technique is utilized. Therein, the spatial displacement field of the microshell can be expressed as [58]

(61) $$u(x,\theta ,t)=U_{mn}\frac{\partial \phi \left(x\right)}{\partial x}\cos(n\theta )e^{i\omega t}$$

(62) $$v(x,\theta ,t)=V_{mn}\phi \left(x\right)\sin(n\theta )e^{i\omega t}$$

(63) $$w(x,\theta ,t)=W_{mn}\phi \left(x\right)\cos(n\theta )e^{i\omega t}.$$

Thereinto, the axial modal function *ϕ*(*x*) is:

(64) $$\phi \left(x\right)=c_{1}\cosh\left(\frac{\lambda_{i}x}{L}\right)+c_{2}\cos\left(\frac{\lambda_{i}x}{L}\right)-\zeta_{i}\left[c_{3}\sinh\left(\frac{\lambda_{i}x}{L}\right)+c_{4}\sin\left(\frac{\lambda_{i}x}{L}\right)\right].$$

The simply supported boundary condition is expressed as

(65) $$\frac{\partial^{2}\phi \left(x\right)}{\partial x^{2}}=\phi \left(x\right)=0\;\left(x=0,L\right).$$

The clamped boundary condition is expressed as

(66) $$\frac{\partial \phi \left(x\right)}{\partial x}=\phi \left(x\right)=0\;\left(x=0,L\right).$$

The free boundary condition is

(67) $$\frac{\partial^{2}\phi \left(x\right)}{\partial x^{2}}=\frac{\partial^{3}\phi \left(x\right)}{\partial x^{3}}=0\;\left(x=0,L\right).$$

Herein, free–simply supported (F–SS), clamped–free (C–F), clamped–simply supported (C–SS), free–free (F–F), and clamped–clamped (C–C) boundary conditions are taken into account. Based on Equations (65)–(67), the constants *c*_1_, *c*_2_, *c*_3_, *c*_4_, *λ_i_*, and *ζ_i_* (*i* = 1, 2, 3, 4…) are given in Table 2. 

Substituting Equations (61)–(63) into Equations (42)–(44) and neglecting axial load $N_{xx}^{0}$, then applying the Galerkin method [59,60,61,62], yields

(68) $$\left(K_{1}-\omega^{2}M_{1}\right)\left\{\begin{array}{c} U_{mn}\\ V_{mn}\\ W_{mn} \end{array}\right\}=\left\{\begin{array}{c} 0\\ 0\\ 0 \end{array}\right\}$$

where **M**_1_ and **K**_1_ represent the mass matrix and stiffness matrix, respectively. By solving the eigenvalue problem, the natural frequencies and eigenvectors are obtained.

## 4. Results and Discussion

In order to verify the correctness of the derivation in this paper, we chose an SS–SS isotropic homogeneous shell to make a comparison without considering the small-scale effect (*l* = 0). The system parameters used are as follows: *ρ* = 2300 kg/m^3^, *ν* = 0.3, *E* = 1.06 TPa, *R* = 2.32 nm, *L*/*R* = 5. As can be seen in Table 3, very good agreement was achieved.

Considering the scale effect, dimensionless natural frequencies of an isotropic homogeneous cylindrical nanoshell were calculated, and are compared with the literature in Table 4. Here the parameters used are the same as those in Table 3. It was found that the present results match those given by Beni et al. [42] well, bespeaking the validity of the present study. 

To make further comparisons, we considered a homogeneous cylindrical shell with the C–C boundary condition. The comparison result is listed in Table 5. It can be seen that the present results agree well with those in the literature [64]. 

Hereinafter, we conducted free vibration and buckling analyses of 3D-GrF cylindrical microshells. If not specified, the geometrical and material properties of the 3D-GrF microshell are:

$$\begin{array}{c} E_{1}=1.02\;\mathrm{TPa},\;\rho_{1}=2300\;\mathrm{kg}/m^{3},\;\nu =0.3\\ l=15\;\mu m,\;m=1,\;h=15\;\mu m,\;R/h=40,\;L/R=2 \end{array}$$

**Figure 3:**
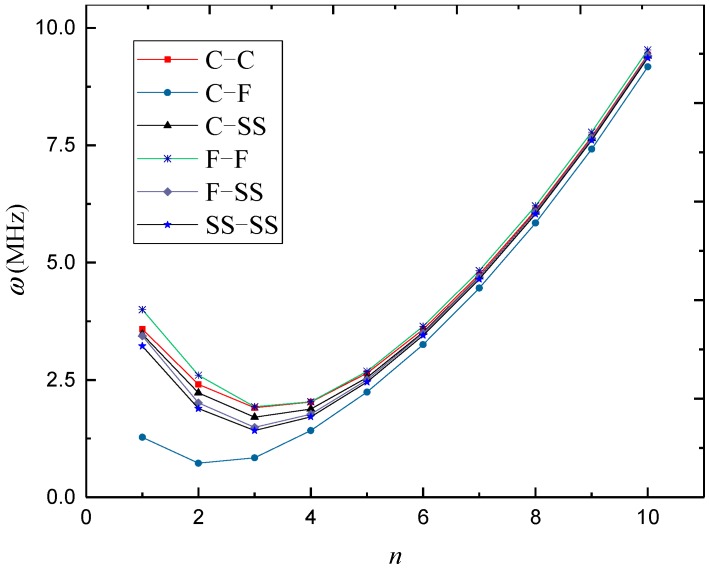
Variation of natural frequency *ω* (MHz) against circumferential wave number *n* of 3D-GrF microshell (3D-GrF-I).

**Figure 4:**
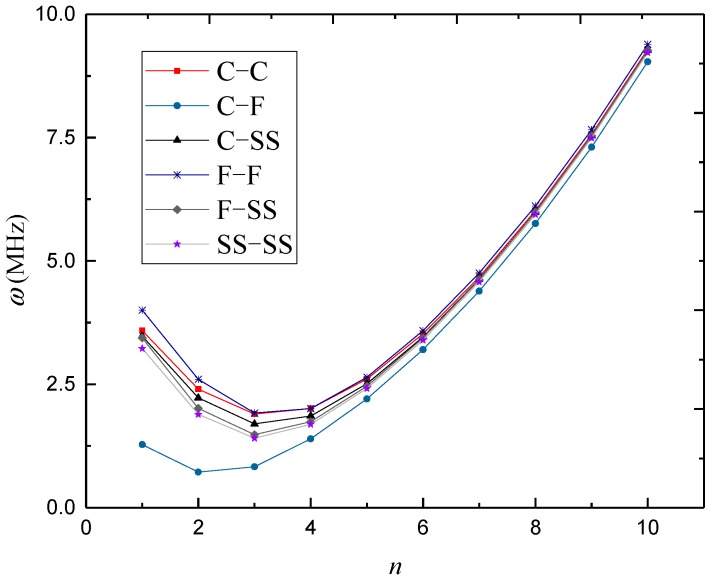
Variation of natural frequency *ω* (MHz) against circumferential wave number *n* of 3D-GrF microshell (3D-GrF-II).

**Figure 5:**
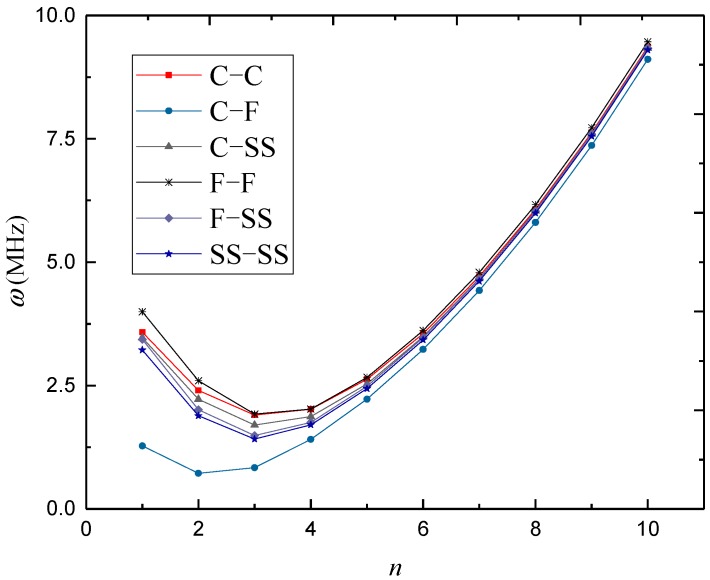
Variation of natural frequency *ω* (MHz) against circumferential wave number *n* of 3D-GrF microshell (3D-GrF-U).

In Figure 3, Figure 4 and Figure 5, natural frequencies of 3D-GrF microshells under various boundary conditions are shown for different foam distributions, where *κ*_0_ = 0.2. For all six of the boundary conditions, one can see the natural frequencies first decreased and then increased as circumferential number *n* increased. The minimum frequency occurred at *n* = 2 for the C–F boundary condition and occurred at *n* = 3 for the other boundary conditions. In addition, the natural frequency of the C–F microshell was the lowest while that of the F–F one was the highest. When the circumferential wave number was greater than 5, natural frequencies under various boundary conditions tended toward the same value. This result shows that the boundary condition effect was closely associated with the circumferential wave number.

**Figure 6:**
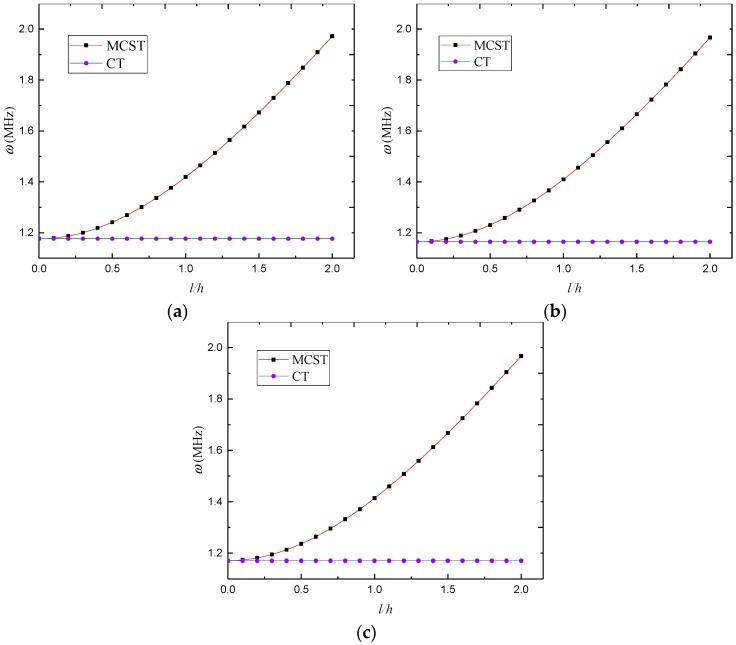
Natural frequency *ω* (MHz) versus dimensionless length scale parameter for SS–SS 3D-GrF microshell: (**a**) 3D-GrF-I; (**b**) 3D-GrF-II; (**c**) 3D-GrF-U.

Based on the modified couple stress theory and classical continuum theory, Figure 6 shows the natural frequency versus dimensionless length scale parameter for the SS–SS 3D-GrF microshell, where *n* = 3 and *κ*_0_ = 0.2. One can see that the natural frequency from the modified couple stress theory increased while that from the classical theory did not change with length scale parameter. This is because size effect tends to increase the stiffness of the 3D-GrF microshell. Nevertheless, the classical shell theory fails to incorporate this effect and thus produces inaccurate results.

**Figure 7:**
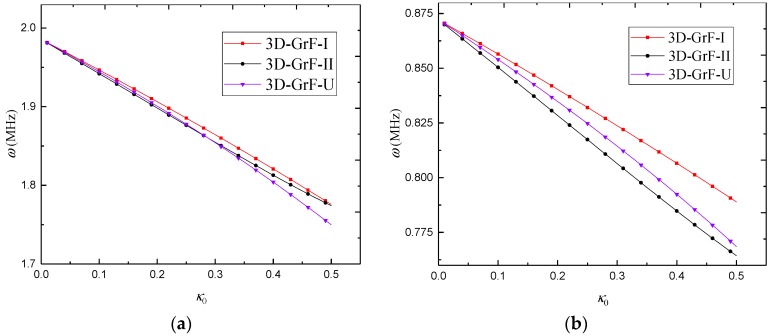

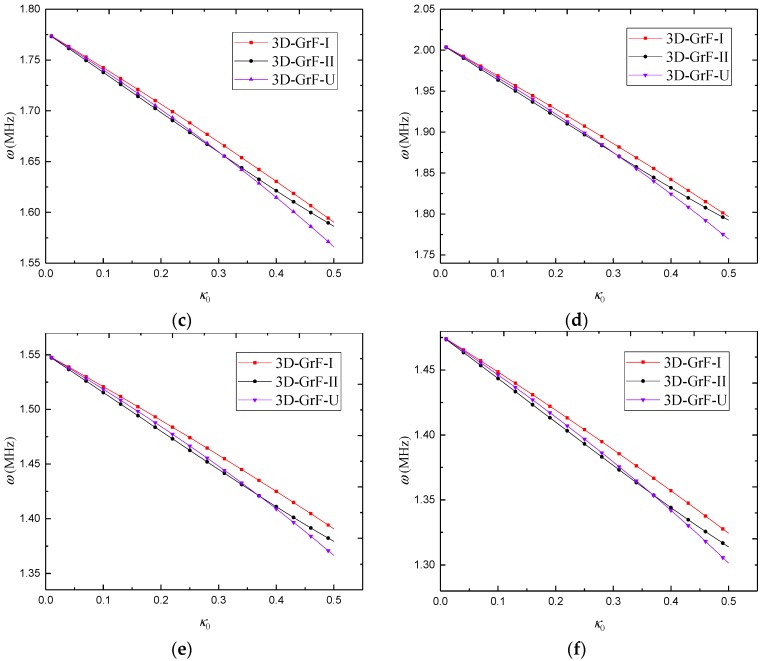
Variation of natural frequency *ω* (MHz) against foam coefficient *κ*_0_ under various boundary conditions (*n* = 3): (**a**) C–C; (**b**) C–F; (**c**) C–SS; (**d**) F–F; (**e**) F–SS; (**f**) SS–SS.

Figure 7 gives the variation of natural frequency against the foam coefficient for different foam distributions. The results show that the increase in the foam coefficient led to a decrease in the natural frequencies of 3D-GrF cylindrical microshells. When the foam coefficient was small, the 3D-GrF-I microshell had the highest natural frequency while the 3D-GrF-II one had the lowest natural frequency. With the increase in the foam coefficient, however, the natural frequency of the 3D-GrF-U microshell dropped faster than that of the 3D-GrF-II one. At last, the 3D-GrF-U microshell had the lowest natural frequency among the three types of foam distribution, except in the C–F boundary condition.

**Figure 8:**
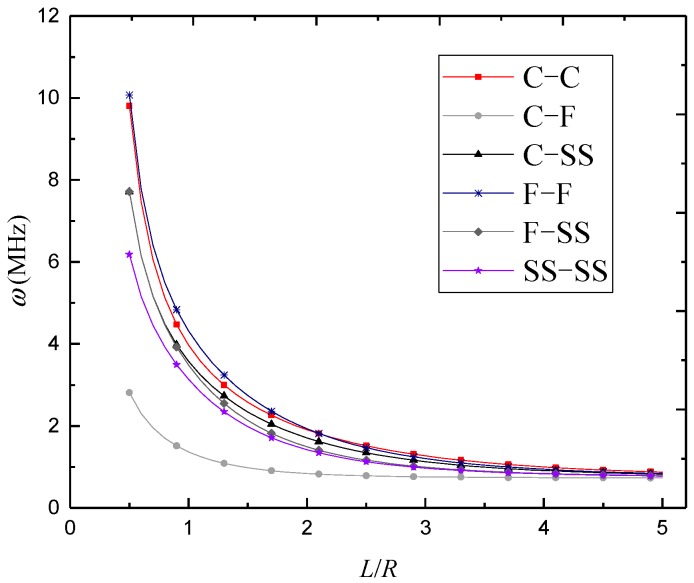
Variation of natural frequency *ω* (MHz) against length-to-radius ratio *L*/*R* of 3D-GrF microshell (3D-GrF-I).

**Figure 9:**
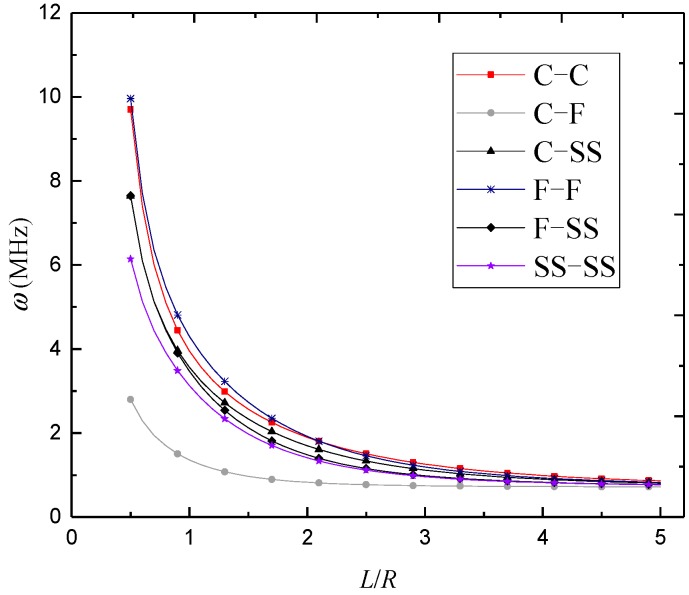
Variation of natural frequency *ω* (MHz) against length-to-radius ratio *L*/*R* of 3D-GrF microshell (3D-GrF-II).

**Figure 10:**
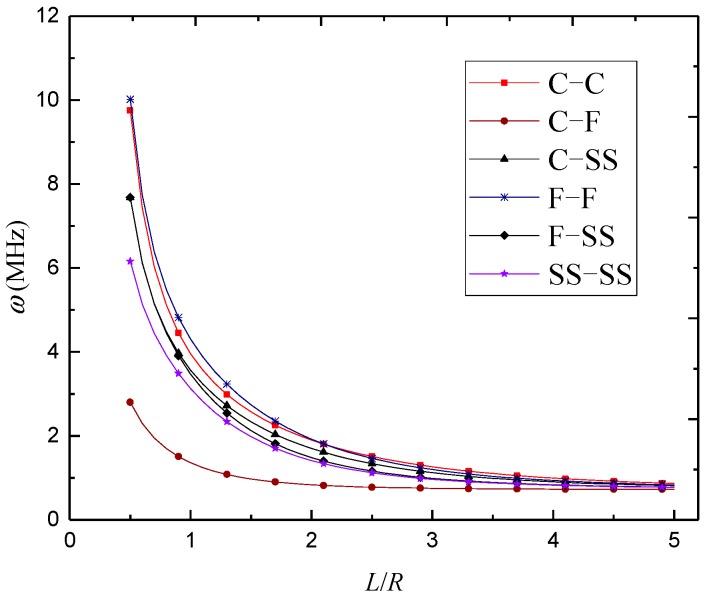
Variation of natural frequency *ω* (MHz) against length-to-radius ratio *L*/*R* of 3D-GrF microshell (3D-GrF-U).

The variations of natural frequency against the length-to-radius ratio for different foam distributions are illustrated in Figure 8, Figure 9 and Figure 10, where *n* = 3 and *κ*_0_ = 0.2. It was found that the natural frequencies kept decreasing with the length-to-radius ratio. It can also be seen that the natural frequencies of 3D-GrF microshells under various boundary conditions tended toward the same value as the length-to-radius ratio increased. 

Table 6, Table 7 and Table 8 show the natural frequency versus radius-to-thickness ratio for different foam distributions, where *n* = 3 and *κ*_0_ = 0.2. It is clear that natural frequencies decreased as the radius-to-thickness ratio rose for all the boundary conditions. 

Table 9 lists the variation of buckling load against circumferential wave number for the SS–SS 3D-GrF microshell, where *L*/*R* = 3 and *κ*_0_ = 0.2. Results show the buckling loads initially decreased and then increased with the circumferential wave number. It can be seen that the critical buckling load *P*_cr_ occurred at *n =* 3. Therefore, mode (*m* = 1, *n* = 3) is chosen as a representative mode in the next buckling studies.

The buckling load against radius-to-thickness ratio for the SS–SS 3D-GrF microshell is listed in Table 10, where different foam distributions are taken into account. It is shown that the larger radius-to-thickness ratio led to the lower buckling load. One can also see that the buckling loads evaluated by the modified couple stress theory were higher than those evaluated by the classical theory, showing that the classical theory underestimated the buckling loads of 3D-GrF microshells. 

**Figure 11:**
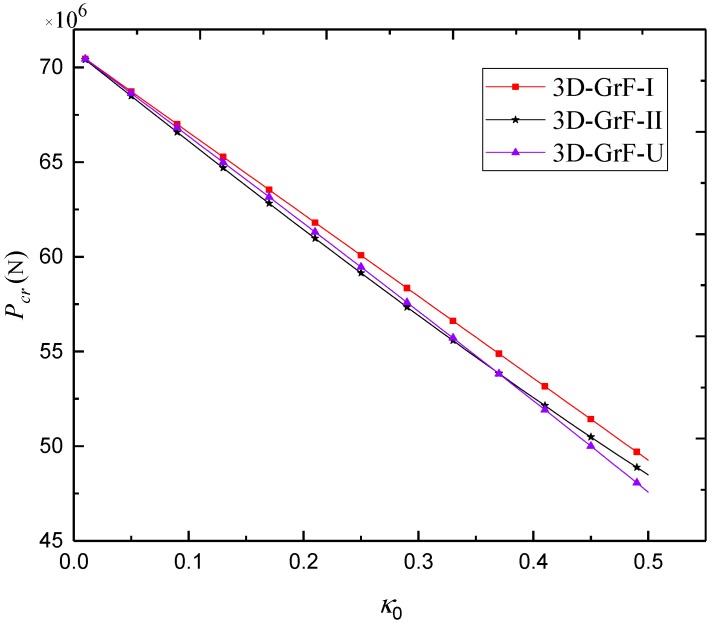
Critical buckling load *P*_cr_ against foam coefficient *κ*_0_ for SS–SS 3D-GrF microshell (*n* = 3).

Figure 11 examines the critical buckling load against the foam coefficient for SS–SS 3D-GrF microshells with different foam distributions. One can see that the higher foam coefficient led to the lower critical buckling load. Additionally, the 3D-GrF-I microshell had the highest critical buckling load. This shows that compact inner and outer surfaces and sparse mid-plane can lead to higher structural stiffness of 3D-GrF microshells. 

**Figure 12:**
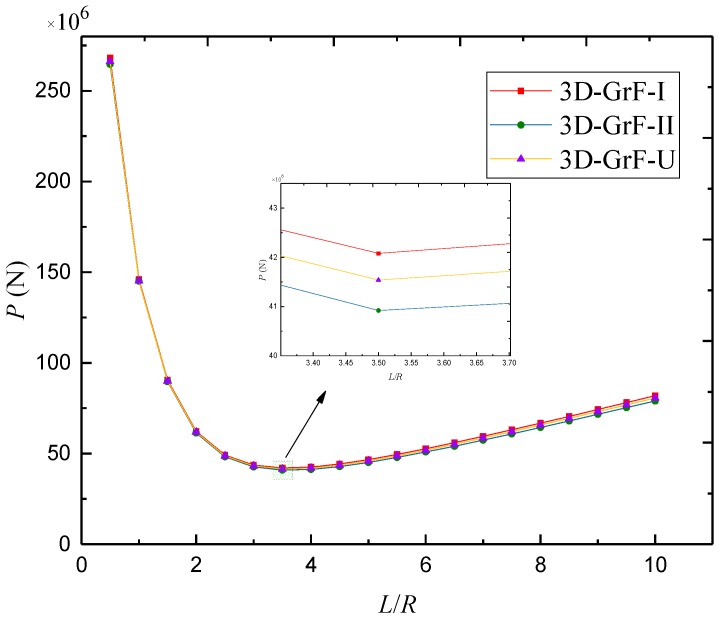
Buckling load *P* against length-to-radius ratio *L*/*R* for SS–SS 3D-GrF microshell (*n* = 3, *κ*_0_ = 0.2).

Figure 12 depicts the buckling load against length-to-radius ratio for SS–SS 3D-GrF microshells with different foam distributions. With the increase of length-to-radius ratio, buckling load decreased initially and then increased. The lowest value of buckling loads occurred at nearby *L*/*R* = 3.5 for all types of foam distribution. 

## 5. Conclusions

In this study, size-dependent free vibration and buckling of cylindrical 3D-GrF microshells were investigated. Formulation of the 3D-GrF microshell was extracted using the modified couple stress theory and the thin shell theory. The Navier method and the Galerkin method were utilized to get critical buckling loads and natural frequencies of 3D-GrF microshells. Results indicate that the foam distribution and foam coefficient play important roles in the buckling and vibration behavior of 3D-GrF microshells. An increase in the foam coefficient led to the smaller natural frequency and critical buckling load. When the foam coefficient was small, the 3D-GrF-I microshell had the highest while its 3D-GrF-II counterpart had the lowest natural frequency and critical buckling load; when the foam coefficient was large, the 3D-GrF-U microshell had the lowest natural frequency, except in the C–F boundary condition. Moreover, compact inner and outer surfaces and sparse mid-plane can the enhance structural stiffness of 3D-GrF microshells.

## Figures and Tables

**Table 1:** Foam coefficients for different distributions.

$\kappa_{0}$	$\kappa_{0}^{*}$	ϑ
0.1	0.1734	0.9360
0.2	0.3426	0.8713
0.3	0.5065	0.8058
0.4	0.6637	0.7391
0.5	0.8112	0.6711
0.6	0.9432	0.6012
0.65	0.9976	0.5653

**Table 2:** Values of *c*_1_, *c*_2_, *c*_3_, *c*_4_, *ζ_i_*, and *λ_i_* for different boundary conditions.

Boundary Condition	*c*_1_	*c*_2_	*c*_3_	*c*_4_	*ζ_i_*	*λ_i_*
C–C	1	−1	1	−1	$\frac{\cosh\left(\lambda_{i}\right)-\cos\left(\lambda_{i}\right)}{\sinh\left(\lambda_{i}\right)-\sin\left(\lambda_{i}\right)}$	$\cosh\left(\lambda_{i}\right)\cdot \cos\left(\lambda_{i}\right)=1$
C–SS	1	−1	1	−1	$\frac{\cosh\left(\lambda_{i}\right)-\cos\left(\lambda_{i}\right)}{\sinh\left(\lambda_{i}\right)-\sin\left(\lambda_{i}\right)}$	$\tan\left(\lambda_{i}\right)=\tanh\left(\lambda_{i}\right)$
F–F	1	1	1	1	$\frac{\cosh\left(\lambda_{i}\right)-\cos\left(\lambda_{i}\right)}{\sinh\left(\lambda_{i}\right)-\sin\left(\lambda_{i}\right)}$	$\cosh\left(\lambda_{i}\right)\cdot \cos\left(\lambda_{i}\right)=1$
C–F	1	−1	1	−1	$\frac{\sinh\left(\lambda_{i}\right)-\sin\left(\lambda_{i}\right)}{\cosh\left(\lambda_{i}\right)+\cos\left(\lambda_{i}\right)}$	$\cosh\left(\lambda_{i}\right)\cdot \cos\left(\lambda_{i}\right)=-1$
F–SS	1	1	1	1	$\frac{\cosh\left(\lambda_{i}\right)-\cos\left(\lambda_{i}\right)}{\sinh\left(\lambda_{i}\right)-\sin\left(\lambda_{i}\right)}$	$\tan\left(\lambda_{i}\right)=\tanh\left(\lambda_{i}\right)$

**Table 3:** Comparison of dimensionless natural frequency Ω($\Omega =\omega R\sqrt{\rho /E}$) of a simply supported–simply supported (SS–SS) isotropic homogeneous cylindrical shell (*κ*_0_ = 0, *l* = 0).

*h/R*	(*m*, *n*)	Present	Beni et al. [42]	Alibeigloo and Shaban [63]
0.02	(1,1)	0.1954	0.1954	0.1968
	(2,2)	0.2532	0.2532	0.2563
	(3,3)	0.2772	0.2772	0.2773
0.05	(1,1)	0.1959	0.1959	0.2004
	(2,2)	0.2623	0.2623	0.2633
	(3,3)	0.3220	0.3220	0.3158

**Table 4:** Comparison of dimensionless natural frequency Ω of an SS–SS isotropic homogeneous cylindrical nanoshell (*κ*_0_ = 0, *l* = *h*).

*h/R*	(*m*, *n*)	Present	Beni et al. [42]
0.02	(1,1)	0.1955	0.1955
	(2,2)	0.2575	0.2575
	(3,3)	0.3067	0.3067
0.05	(1,1)	0.1963	0.1963
	(2,2)	0.2869	0.2869
	(3,3)	0.4586	0.4586

**Table 5:** Comparison of dimensionless natural frequency Ω$\left(\Omega =\omega \cdot R\cdot \sqrt{\left(1-\nu^{2}\right)\rho /E}\right)$ of a clamped–clamped (C–C) isotropic homogeneous cylindrical shell (*κ*_0_ = 0, *l* = 0, *m* = 1, *ν* = 0.3, *h*/*R* = 0.01, *L*/*R* = 20).

*n*	Present	Razavi et al. [64]
1	0.034458	0.033844
2	0.015826	0.015770
3	0.025380	0.024826
4	0.045010	0.045001

**Table 6:** Natural frequency *ω* (MHz) of 3D-GrF microshell with different radius-to-thickness ratios (3D-GrF-I).

*R/h*	SS–SS	C–SS	C–C	C–F	F–F	F–SS
20	4.237	4.818	5.309	3.097	5.442	4.478
30	2.174	2.553	2.835	1.427	2.884	2.289
40	1.419	1.707	1.906	0.840	1.928	1.490
50	1.048	1.281	1.436	0.567	1.447	1.097
60	0.830	1.027	1.154	0.417	1.160	0.869
70	0.689	0.859	0.967	0.326	0.970	0.720
80	0.589	0.739	0.833	0.265	0.835	0.615
90	0.516	0.649	0.732	0.223	0.733	0.538
100	0.459	0.579	0.654	0.192	0.654	0.478

**Table 7:** Natural frequency *ω* (MHz) of 3D-GrF microshell with different radius-to-thickness ratios (3D-GrF-II).

*R/h*	SS–SS	C–SS	C–C	C–F	F–F	F–SS
20	4.182	4.765	5.254	3.045	5.382	4.420
30	2.154	2.534	2.816	1.405	2.863	2.268
40	1.410	1.698	1.898	0.828	1.919	1.480
50	1.043	1.277	1.432	0.560	1.443	1.092
60	0.828	1.025	1.153	0.412	1.158	0.866
70	0.687	0.858	0.966	0.322	0.969	0.718
80	0.589	0.739	0.833	0.263	0.834	0.615
90	0.515	0.649	0.732	0.221	0.733	0.538
100	0.458	0.579	0.654	0.191	0.654	0.478

**Table 8:** Natural frequency *ω* (MHz) of 3D-GrF microshell with different radius-to-thickness ratios (3D-GrF-U).

*R/h*	SS–SS	C–SS	C–C	C–F	F–F	F–SS
20	4.211	4.792	5.281	3.074	5.412	4.450
30	2.164	2.542	2.824	1.417	2.873	2.278
40	1.414	1.701	1.901	0.835	1.922	1.484
50	1.044	1.278	1.433	0.564	1.443	1.094
60	0.828	1.025	1.152	0.415	1.158	0.867
70	0.687	0.857	0.965	0.324	0.968	0.718
80	0.588	0.738	0.832	0.264	0.833	0.614
90	0.515	0.648	0.731	0.222	0.732	0.537
100	0.458	0.578	0.653	0.191	0.653	0.478

**Table 9:** Variation of buckling load *P* (10^7^ N) against circumferential wave number *n* for SS–SS 3D-GrF microshell.

*n*	3D-GrF-I	3D-GrF-II	3D-GrF-U
1	291.546	292.449	291.078
2	13.640	13.617	13.594
3	4.369	4.265	4.318
4	5.521	5.349	5.443
5	8.223	7.966	8.106
6	11.705	11.346	11.541
7	15.858	15.376	15.637
8	20.657	20.036	20.372
9	26.098	25.318	25.740
10	32.180	31.221	31.739

**Table 10:** Buckling load *P* (10^6^ N) against radius-to-thickness ratio *R*/*h* for SS–SS 3D-GrF microshell (*n* = 3, *κ*_0_ = 0.2).

*R/h*	3D-GrF-I		3D-GrF-II		3D-GrF-U	
	CT	MCST	CT	MCST	CT	MCST
30	63.509	109.735	61.314	107.690	62.528	108.682
40	42.734	62.244	41.860	61.433	42.296	61.775
50	32.374	42.365	31.961	41.984	32.133	42.109
60	26.157	31.940	25.942	31.744	26.007	31.780
70	21.996	25.638	21.879	25.532	21.893	25.529
80	19.006	21.446	18.941	21.389	18.930	21.366
90	16.747	18.461	16.713	18.432	16.689	18.400
100	14.978	16.227	14.962	16.215	14.931	16.178

## Appendix A

The non-zero elements in the matrices of Equation (58) are given by

$$K_{1,1}=\frac{D_{5,0}n^{2}\left[4R^{2}+l^{2}\left(4+n^{2}+{k_{m}}^{2}R^{2}\right)\right]}{4R^{4}}+D_{1,0}{k_{m}}^{2};$$

$$K_{1,2}\frac{k_{m}n\left[D_{5,0}l^{2}n^{2}+\left(-4D_{3,0}-4D_{5,0}+D_{5,0}{k_{m}}^{2}l^{2}\right)R^{2}\right]}{4R^{3}};$$

$$K_{1,3}=-\frac{k_{m}\left[2D_{5,1}n^{2}\left(l^{2}+2R^{2}\right)-3D_{5,0}l^{2}n^{2}R+2R^{2}\left(D_{3,1}n^{2}+RD_{3,0}+D_{1,1}{k_{m}}^{2}R^{2}\right)\right]}{2R^{4}};$$

$$K_{2,1}=K_{1,2};$$

$$K_{2,2}=\frac{4D_{1,0}n^{2}R^{2}+D_{5,0}\left\{4{k_{m}}^{2}R^{4}+l^{2}\left[4n^{2}+\left(12+n^{2}\right){k_{m}}^{2}R^{2}+{k_{m}}^{4}R^{4}\right]\right\}}{4R^{4}};$$

$$K_{2,3}=\frac{nD_{5,0}l^{2}\left(2n^{2}+5{k_{m}}^{2}R^{2}\right)+2Rn\left[D_{1,1}n^{2}+D_{5,1}{k_{m}}^{2}\left(l^{2}+2R^{2}\right)+RD_{1,0}+D_{3,1}{k_{m}}^{2}R^{2}\right]}{2R^{4}};$$

$$K_{3,1}=K_{1,3};$$

$$K_{3,2}=K_{2,3};$$

$$\begin{array}{l} K_{3,3}=\frac{1}{R^{4}}[D_{5,0}l^{2}n^{4}+2D_{1,1}n^{2}R+D_{1,0}R^{2}+D_{5,0}{k_{m}}^{2}l^{2}R^{2}+2D_{32}{k_{m}}^{2}n^{2}R^{2}+2D_{50}{k_{m}}^{2}l^{2}n^{2}R^{2}\\ \;+2D_{31}{k_{m}}^{2}R^{3}+D_{50}{k_{m}}^{4}l^{2}R^{4}+D_{52}{k_{m}}^{2}n^{2}\left(l^{2}+4R^{2}\right)+D_{12}\left(n^{4}+{k_{m}}^{4}R^{4}\right)]; \end{array}$$

$$M_{1,1}=-I_{1,0};$$

$$M_{2,2}=-I_{1,0};$$

$$M_{3,3}=-I_{1,0}-I_{1,2}\left({k_{m}}^{2}+\frac{n^{2}}{R^{2}}\right);$$

$$K_{g3,3}=\frac{n^{2}\pi^{2}}{L^{2}}.$$

where $k_{m}=m\pi /L$.
